# Sensors and Advanced Sensing Techniques for Computer Vision Applications

**DOI:** 10.3390/s25010035

**Published:** 2024-12-24

**Authors:** Christos-Nikolaos Anagnostopoulos, Stelios Krinidis

**Affiliations:** 1 Department of Cultural Technology and Communication, University of the Aegean (UAEGEAN), 81100 Mytilene, Greece; 2 Management Science and Technology Department, Democritus University of Thrace (DUTh), 65404 Kavala, Greece

## 1. Introduction and Current Trends in the Field

Computer vision is a multidisciplinary field that enables machines to interpret and understand visual information from the world, simulating human vision. It encompasses a variety of tasks, including object detection, image segmentation, and image understanding, all of which have seen significant advancements due to the integration of Artificial Intelligence techniques. The evolution of computer vision can be traced back to its early days in the 1950s, focusing primarily on two-dimensional image analysis. However, the advent of deep learning, particularly convolutional neural networks (CNNs), has revolutionized the field, allowing for more complex and accurate interpretations of visual data [1,2,3].

Recent studies highlight the transformative impact of deep learning on various applications within computer vision. For instance, object detection has significantly improved using CNNs, which facilitate the identification and localization of objects within images [1,4]. Moreover, image segmentation, a critical aspect of computer vision, has evolved with deep learning methods, enabling precise delineation of objects in images, which is essential for applications ranging from autonomous driving to medical imaging [5]. The integration of CNNs has not only enhanced performance but has also expanded the scope of applications, including real-time systems for precision agriculture and intelligent manufacturing [6,7].

Recent advancements in sensors and advanced sensing techniques have also significantly influenced the field of computer vision, enabling more efficient and effective visual perception systems. One of the most notable trends is the development of in-sensor computing techniques, which allow for data processing directly within the sensor hardware. By processing visual information at the sensor level, systems can significantly reduce the amount of data that needs to be transmitted, thus, enhancing the speed and efficiency of computer vision applications. Recent studies have demonstrated the potential of ferroelectric photosensors for in-sensor artificial neural networks, which can perform computations directly on the sensed data [8,9]. This paradigm shift is particularly beneficial for time-critical applications such as autonomous driving and similar critical decision-making tasks, where rapid decision making is essential.

In addition, the integration of AI into sensor technology is another significant trend. Recent advancements in AI sensors have led to the development of systems that can learn from their environment and adapt to changing conditions [10]. These sensors utilize machine learning algorithms to enhance their performance in tasks such as object detection and recognition. For instance, Complementary Metal Oxide Semiconductor (CMOS) image sensors are enhanced with AI capabilities, allowing them to perform complex computations and improve their accuracy in computer vision tasks [11]. This integration not only improves the functionality of sensors but also expands their applicability across various domains, including healthcare, agriculture, and smart cities.

## 2. Scope of Special Issue and Contributions

The Special Issue on “Sensors and Advanced Sensing Techniques for Computer Vision Applications” aimed to address all topics related to the challenging problems of computer vision and pattern recognition in conjunction with the emerging field of deep learning. As a result of the open call for papers, papers related to deep learning, neural networks, and soft computing have been accepted after a rigorous peer review process and assessed for their technical merit and relevance. The accepted articles cover applied issues in the following fields:

- Deep learning for 2D/3D object recognition and classification;
- Autonomous navigation and robotic agents;
- Data augmentation in computer vision;
- Image fusion, segmentation, and classification from different sensors;
- Parallel Machine Learning;
- Photogrammetry and 3D point clouds;
- Multidisciplinary applications of deep learning, pattern recognition, and computer vision for driving assisting systems and aircraft industry;

More specifically:

In contribution 1, Kumari et al. introduced a method that combines advanced imaging technology (Multi-modal Raman Light Sheet Microscopy) with AI to improve the visualization of complex 3D biological structures and, more specifically, cell cultures and spheroids. Using a special microscopy system, detailed images without needing additional markers are captured, while a deep learning model (Zero-Shot Deconvolution Networks or ZS-DeconvNet) enhances the resolution and sharpness without adding artifacts. This approach provides significant potential for advancing high-resolution imaging in biomedical research and other related fields.

In contribution 2, Trung et al. presented a non-contact method for measuring human height in various postures using computer vision and Deep Learning (MediaPipe library and the YOLOv8 model). By analyzing images from a smartphone camera, the proposed method identifies body joint points with advanced algorithms and calculates height using a regression model. Tested on 166 individuals in different postures, the method achieves high accuracy with minimal error. Future improvements aim to expand its capabilities to more positions and scenarios, increasing its usefulness across healthcare, sports, and other fields.

In contribution 3, Giakoumidis et al. proposed an innovative method (ARM4CH) for automating the 3D modeling of cultural heritage monuments using robotic agents (quadrupedals and UAVs) equipped with advanced sensors. These robotic agents may perform the scanning process systematically and accurately, reducing the need for human expertise and intervention. The approach is designed to improve efficiency and to act a key enabler to applications like digital twins for monitoring and managing cultural sites and spaces. ARM4CH aligns with Industry 4.0 principles and sets the groundwork for future real-world testing.

In contribution 4, Xiang et al. presented a new method for merging infrared and visual images into a single, detailed image by combining their unique features. A specially designed filter (Local Extrema-Driven Image Filter) extracts and processes bright and dark features from both images, which are then fused using advanced techniques. The final image integrates these features along with structural and intensity-based elements, producing superior results. Tests on a standard dataset demonstrate that this method outperforms or at least has equal results compared to eleven state-of-the-art image-fusion methods in terms of quality and accuracy.

In contribution 5, Borstelmann et al. introduced a cutting-edge method to add color to near-infrared (NIR) images, addressing the challenges posed by differences in light properties between NIR and visible light. Traditional methods struggle due to the lack of paired training data, so the researchers use diffusion models, a powerful alternative to Generative Adversarial Networks (GANs). The framework translates NIR intensities into visible light, achieving impressive results. Experiments demonstrate that even simple implementations rival GANs, while more advanced versions outperform them. This work bridges the fields of diffusion models, NIR colorization, and visible-NIR fusion, advancing techniques for biodiversity monitoring, capturing wildlife activities day and night.

In contribution 6, Petracchi et al. focused on accelerating the processing of hyperspectral imaging (HSI), which is widely used in fields like medicine for diagnostics and surgery guidance. To address the challenge of processing large HSI datasets quickly, the researchers parallelized three popular machine-learning algorithms—SVM, Random Forest, and XGBoost—using GPU-based CUDA technology. Results show significant speed improvements, especially for SVM and XGBoost, making them more effective for classifying hyperspectral skin cancer images. The authors illustrate the parallelization techniques adopted for each approach, highlighting the suitability of Graphical Processing Units (GPUs) to enhance HSI applications, when the issue of rapid disease detection is critical.

In contribution 7, Jarahizadeh et al. compared three popular software tools, namely AgiSoft Metashape, PIX4DMapper, and DJI Terra, for processing UAV data to create 3D models of forested areas. Using datasets collected at different flight altitudes and angles, the researchers evaluated the tools in terms of point cloud density, reconstruction quality, computational time, and tree detection accuracy. The results report that AgiSoft and Pix4D produced denser point clouds, but DJI Terra excelled in generating more complete models with fewer gaps, particularly for trees, power lines, and poles. DJI Terra also presented faster processing times and provided more accurate height contours. The overall findings highlight that DJI Terra is a reliable choice for 3D modeling and tree detection in forestry and urban planning applications.

In contribution 8, Matei et al. introduced a method for designing and efficiently implementing 2D Far Infrared Range (FIR) circular filter banks. The filters are created using a frequency transformation of a 1D prototype following a Gaussian shape, designed to meet specific frequency specifications (peak frequency and bandwidth). The resulting filters are accurate and computationally efficient as a result of a factored transfer function and a polyphase structure combined with block filtering. Two types of filter banks—uniform and non-uniform—are developed, with an example demonstrating precise image reconstruction using the uniform filter bank. The proposed example is reported to achieve low computational complexity, making it practical for system-level applications.

In contribution 9, Cardone et al. presented a fast and efficient fuzzy-based framework for segmenting remote sensing images, implemented on a GIS platform. The method uses the Fast Generalized Fuzzy C-means algorithm to detect spatial relationships between pixels and a validity index to determine the optimal number of clusters. The process generates segmented images and a thematic map where pixel classifications are based on their highest membership degree, with reliability estimates provided for each class. Tested on imagery from Naples, Italy, the method produced results consistent with expert analyses while maintaining high computational speed, making it suitable for large-scale, high-resolution datasets.

In contribution 10, Bhatti et al. introduced a cost-effective, automated method for measuring facial skin temperature using a combination of a low-cost thermopile sensor matrix and a 2D image sensor. By fusing temperature and image data through an affine transformation, the system can assign temperature readings to specific facial regions identified via face recognition. Throughout the paper, the advantages of the proposed method are described. A participant study shows that the method achieves accuracy comparable to commercial infrared forehead thermometers, offering a non-contact and precise alternative without requiring manual alignment.

In contribution 11, Ruiz-Beltrán et al. introduced an eye image detection system implemented on an MPSoC (multiprocessor system-on-chip), which includes a block in the programmable logic (PL) to assess the focus quality of the images. The system can discard images that are out of focus during processing. The solution, designed using Vitis High-Level Synthesis (VHLS), works with a 16 MP sensor and processes over 57 fps. Experiments using the CASIA-Iris-distance V4 database show that the system can successfully discard unfocused images, improving efficiency by eliminating up to 97% of blurry images, which reduces the computational load on subsequent processing steps like segmentation and iris pattern extraction. The overall goal of the study is to make iris recognition systems less intrusive and more user friendly.

In contribution 12, Katunin et al. presented a new approach for the automatic quantification of hidden corrosion using image processing of D-Sight images during periodic inspections. The performance of the algorithm was demonstrated through the inspection of a Mi family military helicopter. The nondimensional quantitative measurement introduced in this study was aligned with qualitative analysis by inspectors that performed qualitative analysis, confirming its effectiveness. The proposed method enables the automation of the inspection process and aids inspectors in assessing the extent and progression of hidden corrosion. The results of the study are of great importance to the aircraft industry (and many more), since hidden corrosion remains a major challenge in aircraft maintenance services.

Ultimately, contribution 13 is a review article that studies the latest trends in object detection, recognition, and tracking algorithms for Advanced Driver Assistance Systems (ADASs). ADASs use a range of sensors, including cameras, radars, and lidars, to perceive the environment and detect and track objects on the road, such as vehicles, pedestrians, cyclists, obstacles, and traffic signs. Specifically, Malligere Shivanna et al. survey the latest object detection, recognition, and tracking algorithms used in ADASs, discussing analytically their functionalities and the datasets employed. The review paper also highlights the need for further research in challenging environments, such as those with low visibility or high traffic density and concludes by exploring the future directions for these algorithms in ADASs.

## 3. Conclusions

As Guest Editors, we feel very delighted and satisfied with the final outcome of this Special Issue (SI) and we anticipate that fellow researchers and members of the scientific community will enjoy studying the articles included in it. Moreover, we would like to express our special thanks to the managing team of the Sensors journal for the continuous efforts and support during all the editing stages in this SI, including the initial preparation and planning, as well as the submission and review process of all the candidate manuscripts. Finally, we feel honored to receive outstanding research papers from the contributing authors, and at the same time we are also grateful to the reviewers for their help, their timely feedback, and their valuable comments.
